# Biomarkers in Pediatric Neuropsychiatric Systemic Lupus Erythematosus: A Systematic Review

**DOI:** 10.3390/life15091445

**Published:** 2025-09-15

**Authors:** Evdoxia Sapountzi, Eleni P. Kotanidou, Vasiliki-Rengina Tsinopoulou, Paraskevi Tatsiopoulou, Vaios Dafoulis, Lilian Athanasopoulou, Lampros Fotis, Assimina Galli-Tsinopoulou

**Affiliations:** 1 Outpatient Rheumatology Unit, 2nd Department of Pediatrics, Faculty of Health Sciences, School of Medicine, Aristotle University of Thessaloniki, AHEPA University General Hospital, 54636 Thessaloniki, Greece; 2 2nd Department of Pediatrics, Faculty of Health Sciences, School of Medicine, Aristotle University of Thessaloniki, AHEPA University General Hospital, 54636 Thessaloniki, Greeceagalli@auth.gr (A.G.-T.); 3 1st Department of Psychiatry, School of Medicine, Faculty of Health Sciences, Aristotle University of Thessaloniki, 54636 Thessaloniki, Greece; patatsiopoulou@auth.gr; 4 Department of Child and Adolescent Psychiatry, Hippokrateio General Hospital, 54642 Thessaloniki, Greece; vadafoulis@yahoo.gr; 5 Department of Child and Adolescent Psychiatry, G. Papanikolaou General Hospital, 57010 Thessaloniki, Greece; lilianathan@gmail.com; 6 Division of Pediatric Rheumatology, Department of Pediatrics, NKUA, ATTIKON General Hospital, 12462 Athens, Greece; lafotis@med.uoa.gr

**Keywords:** autoantibodies, neurocognitive dysfunction, neopterin, anti-ribosomal P, anti-ganglioside M1, neuroimaging, aquaporin-4, pediatric lupus

## Abstract

Diagnosis of neuropsychiatric (NP) involvement in pediatric systemic lupus erythematosus (pNPSLE) remains challenging due to NP symptom heterogeneity, lack of specific clinical criteria and validated diagnostic biomarkers, and invasiveness of common diagnostic methods for CNS disease. Although some biomarkers have been identified in adults, their sensitivity, specificity, and clinical applicability in pNPSLE are uncertain. We performed a systematic review according to PRISMA guidelines to evaluate the current evidence on biomarkers associated with pNPSLE. We searched four databases using appropriate search terms for articles in English. After applying our selection criteria, we included 29 studies, from which we manually extracted relevant parameters, including study design, sample size, patient and disease characteristics, biomarker information, and effect sizes. The risk of bias, study quality, and quality of evidence were assessed with standard methods. Most studies had low quality, small samples, and were performed in single centers, which limited the quality of evidence of the biomarkers investigated. Biomarkers showing promising results, with high specificity and sensitivity or predictive value, included CSF neopterin, serum anti-ganglioside M1, a five-biomarker panel including neuronal proteins and anti-ribosomal P, and serum anti-neuronal. Our findings highlight the need for further replication and functional validation studies in pNPSLE.

## 1. Introduction

Nervous system involvement is a severe and heterogeneous manifestation of systemic lupus erythematosus (SLE), categorized in 19 neuropsychiatric (NP) syndromes according to the 1999 American College of Rheumatology (ACR) classification [1], including 12 central nervous system (CNS) and 7 peripheral nervous system (PNS) manifestations. Cognitive dysfunction, mood disorders, seizures, psychosis, and cerebrovascular disease (CVD) are among the most common CNS disorders, while peripheral neuropathy is a common PNS symptom. NP manifestations in SLE, termed NPSLE, vary greatly in frequency, which ranges from 14 to 95%, depending on the definitions used for the classification of symptoms, the methods used for detection, and differences in population characteristics [2,3]. The CNS is involved in the vast majority of patients with NPSLE (93.1%), whereas PNS symptoms occur less frequently (6.9%) [3]. In pediatric populations, NPSLE seems to be as common as in adults; however, children more frequently present NP symptoms early in the disease course, typically within the first 2 years of diagnosis [4]. Moreover, the condition is more severe in children than in adults, often associated with greater disease activity, higher morbidity, and a more aggressive disease course [5]. This greater severity is due to the inherent higher risk of organ damage in pediatric populations and the vulnerability of the still-developing CNS. Accurate and timely diagnosis of NPSLE remains challenging due to the lack of specific clinical criteria and validated diagnostic biomarkers and relies heavily on the exclusion of other similar conditions via clinical assessment, imaging, and laboratory testing. The diagnosis of pediatric-onset NPSLE (pNPSLE) is also hampered by the cost, long duration, and invasiveness of common diagnostic methods to assess CNS disease, including the difficulty in obtaining biospecimens from children, and particularly biospecimens from the CNS. Analysis of cerebrospinal fluid (CSF) samples for inflammatory markers remains the key laboratory assessment for pNPSLE. Such markers include the white blood cell count, protein, oligoclonal bands, and the IgG/albumin CSF ratio. However, their sensitivity and specificity for NPSLE are rather low, both in adults and in children [2].

Various factors involved in the pathogenesis of NPSLE have been examined as biomarkers to aid diagnosis, monitor disease activity, and predict outcomes. These include autoantibodies (e.g., anti-NR2, anti-ribosomal P [anti-RibP], anti-phospholipid [anti-PL] antibodies), cytokines and chemokines (e.g., interleukin-6 [IL-6], interferon-alpha [IFN-α], and C-X-C motif chemokine 10), complement-associated proteins (e.g., C3, C4, and C5b-9), and indicators of neuronal injury and blood-brain barrier [BBB] disruption (e.g., neurofilament light chain, S100b protein, and CSF–serum albumin ratio) [6,7,8]. While several of these biomarkers have been associated with specific NP manifestations in adults, their diagnostic sensitivity, specificity, and clinical applicability in pNPSLE are uncertain. The applicability of such markers in pediatric populations might be difficult, considering that the expression of various markers may be dependent on the developmental stage (e.g., brain development continues until the second decade of life), disease severity (greater in children), and neuroinflammatory profile (different in children). Additionally, applying adult-derived cut-off values in pediatrics may lead to misclassification and to inappropriate clinical decisions. Therefore, it is critical to identify specific biomarkers that can be applied to pediatric patients in order to improve and speed up diagnosis, inform therapeutic strategies, and ultimately improve patient outcomes.

In this study, we aimed to evaluate and synthesize the current evidence on biomarkers associated with pNPSLE, with particular emphasis on their clinical relevance. We thus performed a systematic review of the literature and provide a narrative synthesis of the results.

## 2. Materials and Methods

### 2.1. Systematic Literature Review

We followed the PRISMA guidelines to perform a systematic literature search of articles relevant to pediatric NPSLE and biomarkers in four databases: PubMed, Scopus, Embase, and Google Scholar. The protocol of the review was registered in the Open Science Framework (https://doi.org/10.17605/OSF.IO/TKBP2, accessed on 14 September 2025). A comprehensive search strategy was developed for each database and customized based on the database’s requirements. We used appropriate search terms that included combinations of MeSH terms and free-text keywords. A sample search string for PubMed was as follows: (neuropsychiatric lupus OR neuropsychiatric systemic lupus erythematosus OR NPSLE) AND (biomarkers OR autoantibodies OR cytokines OR chemokines OR complement OR neurofilament light chain OR cerebrospinal fluid biomarkers) AND (pediatric OR child OR adolescent OR juvenile) AND (diagnosis OR prognosis). The search was conducted from inception to June 2025, and the following limits were already applied at the time of the database search: language: English; species: humans; age: <18; study type: exclude case reports. Additional studies were identified by reviewing the reference lists of relevant articles.

### 2.2. Eligibility Criteria

The following inclusion criteria were applied: (1) original peer-reviewed research articles (cohort, case-control, cross-sectional, or longitudinal studies); (2) pediatric patients (≤18 years old); (3) diagnosis of SLE with neuropsychiatric manifestations; (4) assessment of biomarkers associated with NPSLE, including autoantibodies, cytokines, chemokines, complement markers, neurofilament light chain, and CSF biomarkers; (5) studies reporting diagnostic accuracy, association with specific neuropsychiatric symptoms, or prognostic value of biomarkers in pediatric NPSLE.

Studies were excluded if (1) the population included patients older than 18 years or if the population included one or few pediatric patients but no subgroup analysis for these patients was performed; (2) the study design was a case report, case series, or lacked primary data (e.g., review articles); (3) no biomarkers relevant to pediatric NPSLE were studied.

### 2.3. Study Selection

Two independent reviewers (E.S. and E.P.K.) conducted an initial screening of the titles and abstracts of the studies identified through the database search according to the eligibility criteria described in Section 2.2. All disagreements were resolved by A.G.-T. Full-text articles were reviewed to assess final eligibility for inclusion in the review.

### 2.4. Data Collection and Synthesis

The following data were extracted from each included study: first author and publication year, country of study conduct, and study design (e.g., case-control, cross-sectional, and cohort); participant characteristics, including the total number of participants, total number of participants with NP manifestations, number of participants with NP manifestations at SLE diagnosis, age (range and mean/median), and sex distribution (number/percentage of females); disease characteristics, including diagnosis of SLE and diagnostic criteria used, diagnosis of NPSLE and diagnostic methods used (if mentioned), and type and prevalence of NP manifestations present; and biomarker-related information, including type of biomarkers measured and measurement method, and associations between biomarkers and NP manifestations, along with sensitivity, specificity, and diagnostic accuracy if reported.

Relevant information was searched and extracted manually from the main text, tables, figures, and/or supplementary material of each article and is presented in tabular format. Missing or not available information is marked as “Not reported” in the tables. The findings from the included studies were then synthesized in a qualitative manner in the text.

### 2.5. Risk of Bias Assessment

The risk of bias in the included studies was assessed using the Newcastle–Ottawa Scale (NOS) adapted for different study designs. Each study was classified as a cohort, case-control, or cross-sectional study based on its design, as described in the sources. NOS contains the following domains:

- Selection: This domain assesses the representativeness of the study groups, the clarity of case/control/exposed/unexposed definitions, and whether the outcome was present at the start of the study (for cohorts) or if the exposure was ascertained by a validated tool (for cross-sectional). A maximum of 4 stars are given for cohort and case-control studies and a maximum of 5 stars for cross-sectional studies.
- Comparability: This domain evaluates whether the study adequately controlled for confounding factors between the groups compared, either through design (e.g., matching) or statistical analysis (e.g., multivariate regression). A maximum of 2 stars are awarded, one for controlling the most important factor and another for any additional factors. If no explicit control for confounders is stated, 0 stars are given.
- Outcome/Exposure: This domain assesses how outcomes (for cohort/cross-sectional) or exposures (for case-control) were ascertained and the adequacy of follow-up (for cohorts); a maximum of 3 stars are awarded for cohort and case-control studies and a maximum of 2 stars for cross-sectional.

After scoring, the quality of the studies was determined as per the Agency for Healthcare Research and Quality (AHRQ) standards [9].

### 2.6. Statistical Analysis

No statistical analysis was performed on the data obtained from the included studies.

The quality of evidence for each biomarker was assessed using the Grading of Recommendations, Assessment, Development, and Evaluation (GRADE) approach, considering factors such as study limitations, consistency of results, and precision of estimates [10].

## 3. Results

### 3.1. Search Results

**Figure 1:**
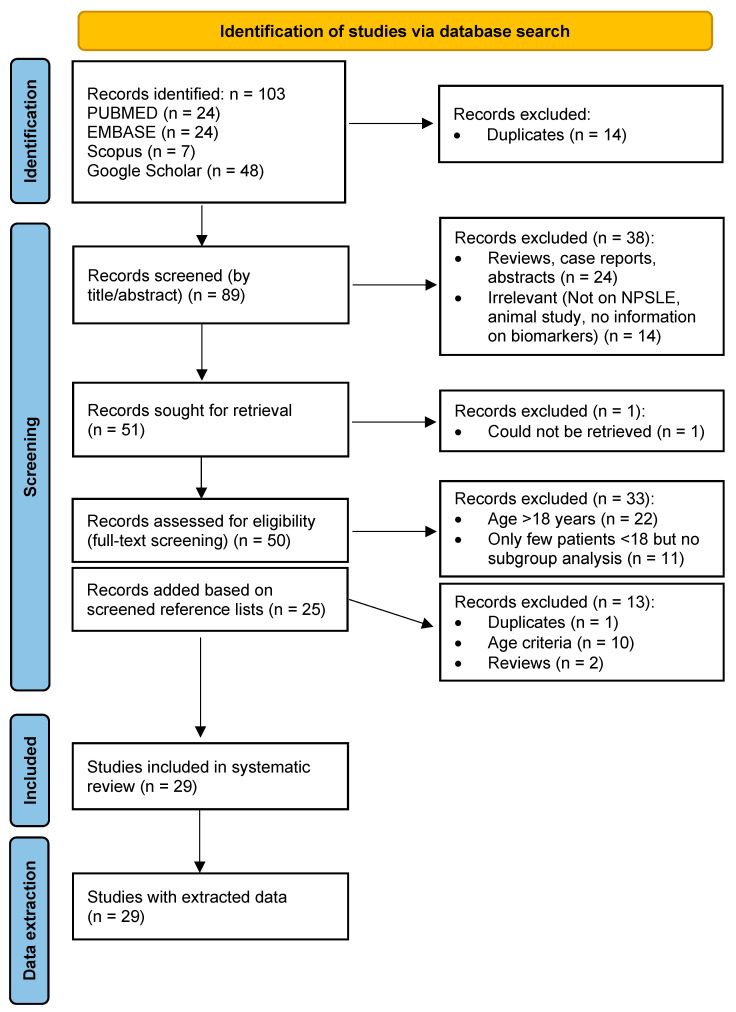
PRISMA flow chart of study selection, along with reasons for excluding studies.

A database search using specific search terms yielded 103 potentially eligible studies, while 25 more studies were identified by reviewing the reference lists of relevant articles. After excluding duplicates and applying our selection criteria, a total of 29 studies were selected for data extraction and narrative synthesis. A PRISMA flow diagram showing the number of studies at each stage of the selection process, along with the reasons for exclusion, is shown in Figure 1.

### 3.2. Study Characteristics

The main study characteristics are shown in Table S1. Of the 29 studies included, 13 were cohort (9 retrospective), 11 were cross-sectional, and 5 were case-control (1 retrospective). Twenty-one studies recruited participants from a single center, five from two centers, and two were multicenter studies. Nine studies reported the follow-up period, with the mean follow-up ranging from 12 months to about 8 years. Six studies were conducted in the United States, five in Egypt, four in China, four in Brazil, two each in France, Canada, the United Kingdom, and India, and one each in Colombia and Iran.

Twenty-one of the twenty-nine studies examined the associations of biomarkers with NPSLE as their primary or one of their main outcomes. The biomarkers tested included autoantibodies in 23 studies, cytokines and inflammatory markers in 7 studies, other proteins (e.g., S100 proteins, neutrophil gelatinase-associated lipocalin [NGAL], neopterin, prolactin, and N-methyl-D-aspartate receptor) in 9 studies, imaging or functional markers in 4 studies, and genetic markers (single nucleotide polymorphisms [SNPs]) in 1 study. Some studies included more than one biomarker category. In most studies that examined protein biomarkers, the method used for biomarker measurement was enzyme-linked immunosorbent assay (ELISA)/digital ELISA; three studies did not report the method used. Other methods used included immunofluorescence or indirect immunofluorescence, double immunodiffusion, immunohistochemistry, flow cytometry, and real-time PCR.

### 3.3. Patient and Disease Characteristics

All analyzed studies included patients with pediatric-onset SLE (pSLE) (diagnosis at <18 years), summing to 2368 pSLE cases, of which 824 were determined to have SLE-related NP manifestations, yielding a prevalence of about 35% (one study did not mention the total number of patients with NP manifestations [11]. However, this result should be interpreted with caution considering that some studies may have included the same patients. Indeed, Labouret et al. (2024) [12] indicated that their sample of 39 patients with pSLE was already described in their earlier study [13]. Moreover, three other studies, performed by the same group [14,15,16], most likely analyzed the same subsets of patients (22, 22, and 15, respectively), recruited in a larger study, judging by the similar patient characteristics and publication year. The majority of the patients were female in all studies (Table S2).

The diagnosis of SLE was established on the basis of the original or revised/updated criteria specified by the ACR in all studies, with 4 studies using the 1982 criteria [17,18,19,20,21], 20 using the 1997 revised criteria [11,15,16,22,23,24,25,26,27,28,29,30,31,32,33,34,35,36,37,38], and 5 using the updated 2019 European League Against Rheumatism/ACR criteria [39]. Diagnosis of NPSLE was performed using the 19 definitions of NP symptoms in the 1999 revised ACR criteria [1] in 15 studies. Formal neurocognitive/neuropsychological testing was performed in six studies.

The NP manifestations reported in the included studies are listed in Table S2. Neurocognitive dysfunction, headache, seizures, mood disorders, anxiety, psychosis, and cerebrovascular disease were among the most common manifestations across studies.

### 3.4. Biomarker Associations with Neuropsychiatric Symptoms

Various SLE autoantibodies were examined for their association with NP symptoms in pediatric patients across the included studies (Table 1 and Table S1). Anti-RibP emerged as the most consistently reported individual autoantibody, showing positive associations with NP symptoms in 5/11 studies, including significantly higher serum levels and significantly higher prevalence rates in pNPSLE than in non-pNPSLE patients. The lowest prevalence in pNPSLE of 25% was reported in a multicenter study in Brazil, including 228 patients [36]. Other studies reported seropositivity in up to 90% of pNPSLE patients [23,24,32,33]. This autoantibody was determined to be highly sensitive for NPSLE (86.7% sensitivity) in a cohort study, albeit with modest specificity (54%) [24]. The remaining six studies showed negative associations with pNPSLE. Other traditional lupus autoantibodies, such as anti-double-stranded DNA (dsDNA), anti-nuclear antibodies (ANA), anti-extractable nuclear antigen (ENA), and anti-PL antibodies, demonstrated negative or conflicting associations across multiple studies, suggesting limited utility for pNPSLE diagnosis (Table 1). A more specialized marker, anti-ganglioside M1, achieved perfect discrimination between pNPSLE and control patients, particularly when combined with anti-RibP (100% predictive value), albeit in a single study [32]. The use of autoantibody clusters was also successful in prediction of pNPSLE compared with individual autoantibodies, with higher prevalence of NP symptoms in patients positive for anti-Smith (Sm), anti-ribonucleoprotein (RNP), and anti-dsDNA [23,27].

Neuronal and brain injury markers were examined in nine studies. Among them, CSF neopterin demonstrated 95% sensitivity and 85% specificity for pNPSLE, while its levels were significantly reduced following treatment. A probability score including CSF neopterin, hallucinations, memory, sleep, and renal involvement was shown to accurately identify pNPSLE [12,13]. Similarly, the use of a panel combining five biomarkers, S100A8/9, S100B, NGAL, anti-NR2, and anti-RibP, achieved 100% sensitivity and 76% specificity for neurocognitive dysfunction among pSLE patients [24]. The combination of anti-neuronal with two autoantibodies (anti-RibP and anti-PL) was highly predictive for psychiatric symptoms [34]. In contrast, the clinical value of these biomarkers when used individually seems to be limited because of negative or inconsistent results or because of very low sensitivity, as in the case of NGAL, despite its high specificity for neurocognitive impairment in pSLE patients [24]. Positivity for antibodies against aquaporin-4 (AQP4), a glial-expressed protein, was associated with neurological involvement, particularly psychosis and seizures [31,40].

Variable results have been reported for other protein biomarkers such as cytokines and chemokines. The possible diagnostic utility of pan-IFN-α levels in the CSF but not in serum was reported in one study, along with a strong correlation with CSF neopterin levels [13]. No significant association with pNPSLE was found for other cytokines like various interleukins, IFN-γ, tumor necrosis factor (TNF)-α, and the soluble TNF receptor 2, and no association was reported for TRAIL and Bcl2 expressed by subpopulations of natural killer cells. Although the protein levels of interleukin (IL)-17 and IL-23 in the serum were not correlated with CNS involvement in pSLE [21], a study reported that SNPs in the genes encoding for the receptors of these interleukins, IL-17RA and IL-23R, respectively, as well as a third polymorphism in Fc gamma receptor 3A (FCGR3A), significantly increase the risk for pNPSLE [42]. One study reported a greater likelihood for pSLE patients with high prolactin levels to present neurological manifestations; however, no direct comparison of patients with and without pNPSLE was performed in the study.

Σε ηωε analysis was used in four studies to identify pNPSLE-specific changes. Structural and functional brain changes were reported in pNPSLE patients, including reduced global and regional gray and white matter volumes [15] and reduced activation in regions involved in working memory and visuoconstructive ability [14]. Moreover, a study using diffusion tensor imaging (DTI) showed decreased global streamline density and connectivity, as well as changes in regional connectivity, in patients with neurocognitive dysfunction [16]. Lastly, a study using magnetic resonance spectrometry (MRS) demonstrated the association between N-acetylaspartate to creatine (NAA/Cr) and choline to creatine (CHO/Cr) ratios with cognitive impairment in pSLE patients [11].

### 3.5. Risk of Bias and Study Quality

Risk of bias assessment using the NOS, adapted for each study design and transformed to AHRQ standards, revealed 4 studies (13.8%) of good quality and 3 studies (10.3%) of fair quality, and the remaining 22 studies (75.9%) were of poor quality, as determined by the selection, comparability, and outcomes/exposure criteria (Table S3). The most common quality issues were single-center recruitment in 21 studies, small sample sizes (<100 patients) in 16 studies, and retrospective design in 10 studies. Selection bias also seemed to be a frequently reported limitation, including underestimation of the prevalence of NP symptoms, selection of more severe cases in tertiary centers, inability to achieve balanced recruitment of patient subgroups, as well as lack of adjustment for possible confounding factors. Although NPSLE was diagnosed using the 1999 ACR criteria for NP classification in most studies (15/29), formal neurocognitive/neuropsychological testing was performed only in 6/29 studies. Therefore, differences in the evaluation of NP symptoms might have differed across studies, and subtle symptoms may have been missed.

Regarding the quality of evidence, the GRADE assessment indicated a very low rating for all biomarkers. Even biomarkers that showed big effect sizes still received low grading because the results were based on single studies with small sample sizes. Conversely, studies with larger samples often included negative associations, thus also lowering the quality of evidence for a given marker.

## 4. Discussion

The lack of objective consensus on NPSLE diagnosis and the highly heterogeneous nature of NP symptomatology in SLE patients pose great challenges in distinguishing these diverse clinical features from those unrelated to SLE. Such challenges are even greater in pediatric patients, who present with even more severe disease. With this systematic review, we aimed to synthesize the available evidence on biomarkers in pNPSLE and evaluate their usefulness in clinical practice by reviewing 29 studies that investigated the value of different serum and CSF markers, genetic signatures, and neuroimaging in classifying SLE and specifically the involvement of the nervous system in pediatric patients.

CSF biomarkers and multiple biomarker panels emerged as being the most clinically significant, demonstrating the best diagnostic performance. CSF neopterin was the best-performing single biomarker, with a 95% sensitivity and 85% specificity across two studies [12,13]. Moreover, its levels were strongly correlated with the CSF levels of IFN-α (R = 0.8323, *p* < 0.0001), and both were reduced after treatment of pNPSLE [13], making them potentially valuable markers for assessing treatment outcomes in these patients. Increased levels of CSF neopterin have been reported in various CNS diseases, including NPSLE, and are considered to reflect CNS inflammation [43], while serum neopterin levels were shown to be elevated in NPSLE than in non-NPSLE in adults [44]. Impressive results were also noted for the five-biomarker panel (S100A8/9, S100B, NGAL, anti-NR2, and anti-RibP) used in the study by Brunner et al. [24], which showed 100% sensitivity and 76% specificity (area under the curve [AUC] 83.4%) for predicting neurocognitive dysfunction in pSLE. Importantly, these five markers were measured in the serum of patients with pSLE, which is a much more accessible biospecimen in children than CSF. It is worth noting that, while some of these markers such as NGAL showed great specificity (92%) for certain aspects of cognitive dysfunction in pSLE, they were not sensitive enough to be used independently (<30% sensitivity), whereas others such as anti-RibP, S100A8/9, and anti-NR2 showed high sensitivity (>85%), but their specificity was low (around 50%), thus limiting their utility as independent markers [24]. A meta-analysis in adult-onset SLE demonstrated a significantly greater proportion of patients with NPSLE being positive for serum anti-NR2 antibodies across 13 studies (pooled odds ratio [OR] = 1.607, 95% confidence interval [CI] 1.041–2.479, *p* = 0.032), although serum levels could not distinguish between different NP syndromes [45]. In contrast, analysis of four studies showed no correlation of the CSF levels of anti-NR2 with NP manifestations. Conversely, the CSF levels of NGAL, also known as lipocalin-2, were shown to be significantly higher in NPSLE than in controls, raising the potential for using CSF NGAL levels as a diagnostic biomarker for NPSLE [46]. The applicability of CSF NGAL in pediatric patients has yet to be examined.

Two other promising combinations of biomarkers emerging from our review were those of anti-neuronal with anti-PL and anti-RibP and of anti-ganglioside M1 with anti-RibP, both of which demonstrated 100% predictive value for pNPSLE [32,34]. Seropositivity for anti-neuronal was found in 70% of pSLE patients with psychiatric disorders but in none of those without such disorders (*p* < 0.0001) [34]. Similarly, seropositivity for anti-ganglioside M1 was found in 83% of patients with pNPSLE and none of the non-NPSLE patients (OR: 36; 95% CI: 4.3–302.8, *p* < 0.001). Thus, a combination of these two highly specific markers with a less specific marker, such as anti-RibP, which, however, shows excellent sensitivity [24], may accurately predict patients with pNPSLE.

Despite being less specific for NP involvement, lupus-specific autoantibodies, including anti-RibP, anti-PL, and anti-dsDNA, as well as other common autoimmune disease markers, like ANA and anti-ENA antibodies, were among the most studied biomarkers in pSLE in the 29 analyzed studies, investigated for their potential association with NPSLE. In adult patients, a previous systematic review found a strong association between serum anti-RibP antibodies and NPSLE (OR: 2.0; 95% CI: 1.2–3.4), particularly psychosis (OR: 3.1; 95% CI: 1.9–4.9) [6]. Our review of pediatric patients revealed mixed results regarding the utility of this marker, with half of the studies showing a significant association, whereas the other half showed no association with pNPSLE. In contrast to the findings in adults, no significant association was shown with pSLE-related psychosis, although anti-RibP could differentiate these children from those with non-SLE-related psychosis [20]. Our NOS analysis revealed that most studies were of poor quality, regardless of sample size, while the effect size (biomarker positivity in pNPSLE vs. non-pNPSLE) varied greatly. Collectively, these findings render the use of anti-RibP in detecting NP symptoms very limited, as also verified by the GRADE assessment.

Anti-dsDNA antibodies are very specific for SLE, correlate with disease activity, and have been reported to have a pathogenic role in SLE. However, they have limited clinical utility in NP involvement, as only about 70% of patients with NPSLE are seropositive, and anti-dsDNA levels do not appear to correlate with NP disease activity [47]. Similarly, we found conflicting results in pediatric populations across studies, with a positive significant correlation of seropositivity with the presence of pNPSLE in one study [29], a negative correlation of anti-dsDNA levels’ elevation with pNPSLE, and even a correlation of higher levels with non-pNPSLE in another study [37], while eight more studies reported no significant correlation with this autoantibody. None of the included studies reported an association of ANA and anti-ENA with pNPSLE.

Anti-PL antibodies, comprising anti-cardiolipin (CL), anti-b2 glycoprotein 1 (b2GP1), and lupus anticoagulant, have been found to be involved in thrombotic events in SLE and have been linked to vascular changes in NPSLE [48], although conflicting results have been published [6]. Moreover, elevations in anti-PL have been associated with some aspects of cognitive function [49], while the presence of anti-PL was shown to be an independent predictor of NP involvement in adults [50]. Another study reported that 57.3% of anti-PL seropositive adult patients had NP manifestations, including CVD, mood disorders, cognitive disorders, seizures, and psychosis as the most frequent ones [51]. In agreement, lupus anticoagulant seemed to increase the risk for NP symptoms in pediatric patients (OR 3.66, 95% CI 1.33–10.3) [38], and a similar prevalence of anti-CL seropositivity of 47.8–57.8% [35,37] and of lupus anticoagulant of 53.8% [35] has been reported in pNPSLE. In contrast, another study showed much lower prevalence (14%) of these antibodies in pNPSLE, which was significantly different from the 57% reported among non-NPSLE patients [12]. Furthermore, Harel et al. [28] reported that anti-CL antibodies are significantly associated with cerebrovascular accidents in pSLE (*p* = 0.03) and that patients testing positive for lupus anticoagulant in two or more tests are more likely to have NP involvement. No association of anti-PL seropositivity with pNPSLE or inconsistent results were reported by most of the other studies that examined this marker. These conflicting results suggest either differences in study designs, population characteristics, and methods to measure anti-PLs or that the overall clinical value for anti-PL is rather low. Regardless, further studies in larger samples are required to assess the diagnostic/screening utility of these autoantibodies.

To explore the clinical value of autoantibody-based subgroup profiling, two of our analyzed studies [23,27] used a two-step cluster approach based on the seropositivity of patients for various autoantibodies (anti-CL, ANA, anti-b2GP1, anti-dsDNA, anti-RNP, anti-RibP, anti-ENA [including anti-Sm, anti-Sjogren’s syndrome (SS)A, and anti-SSB], anti-scl70, and Jo-1). A higher prevalence of NP symptoms was found in clusters characterized by anti-RNP and anti-Sm seropositivity (hazard ratio 6.562, OR (95% CI) 2.274~18.940) [23] and those additionally characterized by anti-dsDNA seropositivity (*p* = 0.036) [27]. Both these studies support the notion that biomarker combinations, and in this case, full autoantibody profiling, may prove more useful in pNPSLE diagnosis/prognosis than individual markers.

A potentially interesting biomarker for pNPSLE is AQP4 antibodies. Two studies examined their involvement in pSLE. One reported seropositivity in about 4% of the patients [31], whereas the other reported 62% positivity [40]. This discrepancy may be due to the different methods used to measure these antibodies, a cell-based assay in the first case and the much more sensitive ELISA in the second. Regardless, both studies reported significantly higher frequency of neurological involvement among AQP4 seropositive patients, and particularly psychosis and seizures. Although these are promising results, both studies compared AQP4-positive with AQP4-negative patients and did not examine AQP4 positivity between pNPSLE and non-pNPSLE patients. Therefore, additional studies are needed to evaluate the clinical utility of this marker for NP involvement in pediatric patients.

While protein biomarkers like those mentioned above may identify patients with established neurological involvement, genetic profiling may enable earlier risk assessment. Although the genetic aspects of NPSLE are only recently being explored, we found one study in pediatric patients with SLE reporting three SNPs in genes regulating immune responses, namely, FCGR3A, IL-17RA, and IL-23R, which significantly increased the risk for pNPSLE development (ORs ranging 4.50–7.83, *p* < 0.05) [42]. In agreement, a previous meta-analysis also demonstrated a significant association between NPSLE and an FCGR3A polymorphism (OR 1.89, 95% CI 1.08–3.30, *p* = 0.03) [52]. Both IL-17A and IL-23R polymorphisms have been reported to increase susceptibility to SLE [53,54]; however, their association with pNPSLE and their utility in NP risk prediction have yet to be verified by future studies.

Beyond protein and genetic biomarkers, neuroimaging markers have also been explored for their association with NPSLE, given their specificity in detecting nervous system abnormalities. Our literature search in pediatric patients revealed four eligible studies exploring such neuroimaging biomarkers for pNPSLE, which consistently demonstrated objective structural and functional brain changes in pNPSLE. MRS revealed significant metabolic alterations in the brain of patients with pNPSLE, with reduced NAA/Cr ratios significantly associated with depressive symptoms (*p* = 0.021), psychosis (*p* = 0.015), chorea (*p* = 0.039), and cognitive impairment (*p* = 0.003) [11]. Additionally, elevated CHO/Cr ratios correlated with CVD (*p* = 0.046) and cognitive impairment (*p* = 0.001) [11]. Structural magnetic resonance imaging (MRI) studies demonstrated reduced global gray and white matter volumes in patients with neurocognitive dysfunction, with particularly notable grey matter volume reductions in the lateral frontal, orbitofrontal, anterior cingulate, and lateral temporal areas, as well as visual association regions, and white matter volume reductions in the anterior corpus callosum, left temporal lobe, dorsal frontal corona radiata, and frontal pole [15]. Functional MRI revealed altered brain activation patterns during cognitive tasks, with patients showing lower activation in the precuneus and inferior parietal regions during working memory tasks and in the precuneus and right occipital regions during visuospatial attention tasks [14]. DTI further supported these findings, showing decreased global streamline density and connectivity in pNPSLE patients with neurocognitive dysfunction (*p* = 0.001 and *p* = 0.013, respectively), with regional connectivity changes affecting frontal, precentral, postcentral, and cerebellar regions [16]. These pediatric neuroimaging findings are consistent with some studies in adult SLE/NPSLE patients, where structural and functional brain abnormalities have been documented [47,55]. Adult studies have similarly demonstrated white matter hyperintensities, brain atrophy, and microstructural damage using advanced neuroimaging techniques. Diffusion tensor imaging studies in adults show reduced fractional anisotropy and increased diffusivity in key brain regions, including the corpus callosum, frontal white matter, and thalamus [56]. Although these structural and connectivity abnormalities may be more frequent in NPSLE than in controls, a number of studies have also reported similar findings in non-NPSLE patients, as well as no changes in patients with a definite diagnosis of NPSLE [55], therefore raising doubt regarding their specificity for NPSLE-related changes.

## 5. Limitations and Future Perspective

While some of the above-described findings are promising, they remain exploratory and require further replication and functional validation. Importantly, most studies were performed in a single center and had small sample sizes, while sample representativeness and comparability were low overall, with only a few exceptions, as determined by NOS scoring. Moreover, GRADE assessment indicated low quality of evidence for all mentioned biomarkers owing to poor study quality for most studies. Even promising biomarkers such as CSF neopterin and anti-ganglioside M1 could not overcome quality limitations, given that the evidence was only obtained from a single study. Moreover, these biomarkers lack validation in large, multicenter cohorts, representative of real-world clinical settings.

Beyond study quality limitations, practical barriers in pediatric practice limit the ability of clinicians to implement these biomarkers in routine care, as performance characteristics may vary significantly across different cohorts and laboratory settings. The most accessible marker, anti-RibP, could serve as an initial screening tool, but advanced assays like CSF neopterin require specialized expertise and equipment not widely available in general pediatric settings. Cost-effectiveness analyses are lacking, and the feasibility of comprehensive biomarker panels is limited by sample volume constraints in children and the need for age-specific reference ranges. A pragmatic implementation approach would prioritize validation of anti-RibP in routine laboratories while establishing referral networks for specialized testing, rather than attempting immediate widespread implementation of complex multi-biomarker panels.

The clinical implementation of these markers is further hampered by the fact that most of the analyzed studies provide proof-of-concept data rather than the robust evidence needed for developing clinical guidelines. Thus, these novel biomarkers may be promising but require high-quality replication in future studies. Specifically, future research should prioritize the following: (1) large, prospective, multicenter validation studies using standardized NPSLE definitions; (2) comparative effectiveness studies evaluating biomarker combinations; (3) cost-effectiveness analyses; and (4) implementation studies assessing real-world performance. Moreover, research in pNPSLE should take advantage of the range of studies published in adults and assess whether emerging biomarkers with satisfactory ability to discriminate between NPSLE and non-NPSLE can be applied to pediatric patients. Such biomarkers include serum IL-6 (AUC 0.89), high-mobility group box protein 1 (AUC: 0.84; *p* < 0.05), CSF-Klotho (AUC: 0.94; *p* < 0.001), various immune complex-associated antigens in the CSF, as well as genetic (SNPs in TREX) and epigenetic (microRNAs miR-23a and miR-155) markers [6]. Challenges faced by clinicians to advance such research pertain to limitations in assembling large pediatric study samples that have a homogeneous representation of NPSLE syndromes, which also preclude subgroup analysis to detect associations of biomarkers with specific NP syndromes. This is additionally burdened by the many inconsistencies in NPSLE classification and terminology used. Moreover, although CSF samples are most appropriate for CNS damage, their collection from pediatric patients is an invasive procedure and a challenging task for doctors. Therefore, future studies should aim to identify markers in more accessible biological samples, exploiting the technological advances in proteomics and next-generation sequencing approaches.

## 6. Conclusions

Collectively, our systematic review reveals that very few biomarkers have actual clinical applicability in pNPSLE, considering the many inconsistencies reported across studies. Biomarkers showing promising results, with high specificity and sensitivity or predictive value, include CSF neopterin, serum anti-ganglioside M1, the five-biomarker panel, including neuronal proteins and anti-RibP, and serum anti-neuronal. Neuroimaging markers such as NAA/Cr and CHO/Cr ratios also show some promising results. Our findings suggest that optimal pNPSLE diagnosis requires a multi-modal approach combining CSF markers, when clinically appropriate, autoantibody profiling, and neuroimaging, as well as using multi-biomarker panels rather than relying on traditional single-marker testing approaches. Importantly, our study highlights the need for additional validation studies in larger samples to develop diagnostic algorithms that incorporate these approaches. Our review sets the basis for such studies while informing clinicians on the advantages and disadvantages of available biomarkers in pNPSLE.

## Figures and Tables

**Table 1:** Biomarker associations with neuropsychiatric symptoms.

Biomarker(s) Analyzed	Key Findings (References)	Clinical Utility Assessment
Autoantibodies		
Anti-RibP	Seropositivity in 50–89% of pNPSLE vs. 0–48% of non-pNPSLE (*p* < 0.05, [23,24,32,33]) Higher frequency of acute confusional state (13% vs. 5%; *p* = 0.041) and mood disorder (18% vs. 8%; *p* = 0.041) among seropositive patients [36] Non-significant association with psychosis (seropositivity in 38% of patients with pSLE-related psychosis vs. 16.6% without psychosis (*p* = 0.085, [20]) Serum levels significantly higher in pNPSLE [25,32] and associated with Ped-ANAM scores (*p* < 0.05, [24]) No significant association with pNPSLE reported in other studies [12,13,27,34,36]	Early diagnosis of pNPSLE; Screening utility: AUC > 0.7, 86.7% sensitivity, 54% specificity Low sensitivity (0.38) and questionable specificity for detecting psychosis among pSLE patients.
Anti-dsDNA	Seropositivity in 82.9% with pNPSLE vs. 66.7% of non-pNPSLE (*p* < 0.05, [29]) Negative correlation of anti-dsDNA elevation with NP symptoms (*p* = 0.022, [18]; 32.6% in pNPSLE vs. 90.9% in non-pNPSLE, *p* < 0.0001, [37]) Levels higher in non-pNPSLE (*p* = 0.001, [37]) No significant association reported in eight studies [11,12,13,20,26,32,33,38]	Conflicting results across studies, low clinical utility in pNPSLE (if any)
Anti-NR2	Positive association with psychomotor speed in pSLE Negative correlation with changes in performance on working memory tests from baseline to follow-up [24]	86.7% sensitivity, 51% specificity for cognitive impairment in pSLE patients
ANA	ANA titer ≥ 1:280 negatively correlated with pNPSLE (*p* = 0.001, [37]) No significant association reported in eight studies [11,12,13,18,26,29,32,38]	Low utility for pNPSLE
Anti-PL	Anti-PL (all): No significant association with pNPSLE reported in three studies [19,26,34] Anti-CL: Significantly associated with CVA in pSLE (*p* = 0.03; [28]) Mixed results for seropositivity: - lower in pNPSLE in one study (14% vs. 57% without pNPSLE, *p* = 0.01, [12]) - higher in pNPSLE in two studies (47.8–57.8% of pNPSLE vs. 19.4–23% nonpNPSLE, *p* = 0.026, [35,37]) - no association in five studies [11,13,18,32,38] Anti-b2GP1: Negative association with overall score and scores in subsets of Ped-ANAM (*p* < 0.05, [24]) No association with pNPSLE reported in three studies [12,13,38] Lupus anticoagulant: Negative association with scores in Ped-ANAM subsets (*p* < 0.05, [24]) Positivity on two or more occasions associated with NP involvement (16% vs. 1%; *p* = 0.01); no significant association with one positive test [28] Non-significant positivity reported in pNPSLE (53.8% vs. 34.7% of non-pNPSLE; [35]) Risk factor for pNPSLE: OR 3.66, 95% CI 1.33–10.3, *p* = 0.01 [38]	Anti-CL: Insight for pathogenic mechanism in pSLE. Low utility as biomarker for pNPSLE Anti-b2GP1: Low utility, mostly no associations reported Lupus anticoagulant: Low diagnostic value, repeated testing limitations; potential use as a risk predictor
anti-ENA	No significant association with pNPSLE reported in six studies [11,12,13,18,26,38]	No utility
Anti-ganglioside M1	Serum IgM levels higher in pNPSLE (*p* < 0.001); no difference in serum IgG levels [32] Seropositivity associated with cognitive dysfunction (83.3% of pNPSLE versus 0% of non-pNPSLE, OR: 36; 95% CI: 4.3–302.8, *p* < 0.001) [32]	High predictive value for pNPSLE and cognitive dysfunction; 100% predictive value when combined with anti-RibP
Autoantibody Clusters		
Cluster 1: anti-dsDNA, low prevalence of other autoantibodies; Cluster 2: anti-SSA/SSB, anti-dsDNA, anti-chromatin, anti-RibP, anti-U1RNP, anti-Sm; Cluster 3: anti-dsDNA, anti-U1RNP, anti-Sm	Higher NP prevalence in cluster 3 (32.8% vs. 19.1% in group 1 and 10% in group 2), *p* = 0.036 [27]	Complete autoantibody profile may help predict the clinical course of pNPSLE
Cluster 1: anti-Sm/RNP^pos^ Cluster 2: anti-Sm/RNP^neg^	More NPSLE in cluster 1 (*p* = 0.001), HR 6.562 OR (95% CI) 2.274~18.940 [23]	Clustered autoantibodies linked to pNPSLE
Neuronal or brain injury markers		
Anti-neuronal	Seropositivity associated with psychiatric disorder (70% versus 0%, *p* < 0.0001, [34])	Potential value in predicting psychiatric symptoms. High predictive value (100%) if combined with anti-PL and anti-RibP
NGAL	Negative association with psychomotor speed. Negative correlation with changes in performance on psychomotor speed tests from baseline to follow-up. [24]	92% specificity; very low sensitivity (26.7%) (not useful alone)
NMDAR	No correlation of serum levels with any measure of cognitive function [33]	No utility as a biomarker in pNPSLE
S100A8/9	Gain in working memory associated with level increases over time (*p* < 0.05, [24])	Considered clinically irrelevant
S100B	Weak negative correlation with measures of cognitive function in pNPSLE patients. Improved accuracy in Ped-ANAM subtests associated with level increases over time (*p* < 0.05, [24]) Association with NP manifestations (*p* < 0.001; OR: 2.3; 95% CI: 1.1–4.3) and cognitive impairment (*p* = 0.03; OR: 3.7; 95% CI: 1.2–7.1) [19] No significant association with cognitive impairment reported in one study [11]	Low utility, inconsistent results
AQP4-IgG	AQP4-IgG positivity in 17.9% of patients with neurological involvement versus 0% of those without (*p* = 0.002, [31]) Higher frequency of neurological disorder (*p* = 0.001), particularly psychosis (*p* = 0.009) and seizures (0.032) in AQP4-seropositive patients [40]	Potential role in patient screening and identification of patients at risk for neurological involvement
CSF neopterin	Higher levels in active pNPSLE versus inactive pNPSLE (*p* = 0.0015) and versus non-pNPSLE patients (*p* = 0.0008). Higher levels at diagnosis than after treatment [13]. Elevated in 73% pNPSLE. Addition of neopterin to a probability score including hallucinations, memory, sleep, and renal involvement correctly identifies pNPSLE [12].	Diagnostic and activity biomarker for pNPSLE Sensitivity: 95%, Specificity: 85%
Set of 5 biomarkers (S100A8/9, S100B, NGAL, anti-NR2, anti-RibP)	AUC: 83.4% (95% CI 73% to 94%). Propensity score of 11 or above [24]	100% sensitivity, 76% specificity for NCD diagnosis
Cytokines/Chemokines		
sTNFR2	No significant association [25]	No utility for pNPSLE
Pan-IFN-α	CSF levels higher in active pNPSLE versus inactive pNPSLE (*p* = 0.0010), no difference to controls. Higher at diagnosis than after treatment. Strong correlation with CSF neopterin (R = 0.8323, *p* < 0.0001). No association of pNPSLE with serum pan-IFN-α [13].	Possible diagnostic utility for CSF levels
Peripheral IFN-γ and TNF-α, IL-17, IL-23	Non-significant correlation of IFN-γ and TNF-α expression levels with CNS involvement (*p* = 0.068 and 0.061, respectively). No correlation with IL-17 and IL-23 [21].	Unknown; needs to be assessed further
IFN-γ, TNF-α, IL-4, -5, -6, -10, -12, and -17	No association with cognitive impairment [11]	No utility for pNPSLE
NK cell populations	Absence of NP symptoms associated with significantly lower TRAIL- and Bcl2-expressing NK cells. Similar cell numbers between patients with NP symptoms and controls [30]	No utility for pNPSLE
Other		
Prolactin	Neurological manifestations more common in patients with hyperprolactinemia (*p* = 0.007) [41]	Unknown; more studies required
Genetic markers		
SNPs FCGR3A rs396991, IL-17RA rs2895332, and IL-23R rs10889677	The CA/CC genotypes of FCGR3A rs396991, GA/AA genotypes of IL-17RA rs2895332, and CA/CC genotypes of IL-23R rs10889677 increase the risk for pNPSLE: (rs396991: OR 5.00, 95% CI 0.99–25.17, *p* = 0.029; rs2895332: OR 7.83, 95%CI 1.47–41.79, *p* = 0.017; rs10889677: OR 4.50, 95%CI 1.08–18.69, *p* = 0.027) [42]	Potential screening utility but conflicting literature results; more studies required
Neuroimaging markers		
MRS metabolites	Reduced NAA/Cr ratio associated with depressive symptoms (*p* = 0.021), psychosis (*p* = 0.015), chorea (*p* = 0.039), and cognitive impairment (*p* = 0.003). CHO/Cr ratio associated with CVD (*p* = 0.046) and cognitive impairment (*p* = 0.001) [11].	NAA/Cr and CHO/Cr may be useful biomarkers for pNPSLE
MRI (gray/white matter)	Reduced global gray and white matter volume in patients with NCD (*p* = 0.0031, 0.065, respectively). Reduced gray matter volume in the lateral frontal, orbitofrontal, anterior cingulate, and lateral temporal areas, as well as visual association regions (*p* < 0.05). Reduced white matter volume in the anterior corpus callosum, left temporal lobe, dorsal frontal corona radiata, and frontal pole (*p* < 0.05) [15].	Morphometric parameters could serve as outcome measures of pNPSLE studies on etiology and treatment
Brain activation during fMRI tasks and during performing FNTs	Lower brain activation during the working memory (precuneus, inferior parietal regions) and VCA (precuneus, right occipital regions) fMRI tasks in patients with NCD (*p* < 0.05). Higher activation during the CPT-IP task (*p* = 0.05). Correlation of activation of the ROIs stimulated by the attention (CPT-IP) and VCA fMRI tasks with the attention and VCA domain z-scores. Negative correlation of working memory performance with activation in ROIs stimulated by the CPT-IP task [14].	fMRI as candidate imaging biomarker for pSLE-associated NCD
Diffusion tensor imaging (streamline density, pairwise connectivity)	Decreased global streamline density and connectivity in patients with pSLE-NCD (*p* = 0.001, 0.013, respectively). Changes in regional connectivity: decreased for pSLE-NCD in frontal, precentral, postcentral, and cerebellar regions; increased in ventral regions, including the right fusiform gyrus and temporal poles [16].	

Abbreviations: ANA, antinuclear antibodies; AQP4, aquaporin-4; AUC, area under the curve; Bcl2, B-cell lymphoma 2, CHO, choline-based; CI, confidence interval; CL, cardiolipin; CPT-IP, continuous performance task-identical pairs; Cr, creatine-containing; CSF, cerebrospinal fluid; CVA, cerebrovascular accident; ds, double-stranded; ENA, extractable nuclear antigens; fMRI, functional MRI; FNT, functional neurocognitive tests; GP1, glycoprotein 1; HR, hazards ratio; IFN, interferon; IL, interleukin; IL-17RA, interleukin 17 receptor A; IL-23R, interleukin 23 receptor; MRI, magnetic resonance imaging; MRS, magnetic resonance spectroscopy; NAA, N-acetylaspartate; NCD, neurocognitive dysfunction; neg, negative; NGAL, neutrophil gelatinase associated lipocalin; NK, natural killer; NMDAR, N-methyl-D-aspartate receptor; NP, neuropsychiatric; NR2, NMDA receptor 2; OR, odds ratio; Ped-ANAM, Pediatric Automated Neuropsychological Assessment Metrics; PL, phospholipid; pNPSLE, pediatric-onset neuropsychiatric SLE; pos, positive; pSLE, pediatric-onset SLE; RibP, ribosomal P; RNP, ribonucleoprotein; ROI, region of interest; S100, S100 calcium binding protein; Sm, Smith; SNP, single nucleotide polymorphism; SSA/SSB, Sjogren’s syndrome A/B; sTNFR2, soluble TNF receptor 2; TNF, tumor necrosis factor; U1RNP, U1 ribonucleoprotein; VCA, visuoconstructive ability.

## Data Availability

No new data were created or analyzed in this study.

## Supplementary Materials

The following supporting information can be downloaded at: https://www.mdpi.com/article/10.3390/life15091445/s1, Table S1: Characteristics of the 29 included studies; Table S2: Patient and Disease Characteristics; Table S3: Newcastle–Ottawa Scale Scoring of Studies and Quality Assessment as per the AHRD Standards.

## Abbreviations

The following abbreviations are used in this manuscript:

ACR	American College of Rheumatology
AHRQ	Agency for Healthcare Research and Quality
ANA	anti-nuclear antibodies
AQP4	aquaporin 4
AUC	area under the curve
BBB	blood-brain barrier
b2GP1	b2 glycoprotein 1
CHO/Cr	choline to creatine
CI	Confidence interval
CL	cardiolipin
CNS	central nervous system
CSF	cerebrospinal fluid
CVD	cerebrovascular disease
dsDNA	double-stranded DNA
DTI	diffusion tensor imaging
ENA	extractable nuclear antigen
ELISA	enzyme-linked immunosorbent assay
FCGR3A	Fc gamma receptor 3A
GRADE	Grading of Recommendations, Assessment, Development, and Evaluation
IFN-α	interferon-alpha
IL	interleukin
MRI	magnetic resonance imaging
MRS	magnetic resonance spectrometry
NAA/Cr	N-acetylaspartate to creatine
NGAL	neutrophil gelatinase-associated lipocalin
NOS	Newcastle–Ottawa Scale
NP	neuropsychiatric
NPSLE	neuropsychiatric systemic lupus erythematosus
NR2	N-methyl-D-aspartate receptor 2
OR	odds ratio
pNPSLE	pediatric-onset neuropsychiatric systemic lupus erythematosus
PL	phospholipid
PNS	peripheral nervous system
pSLE	pediatric-onset systemic lupus erythematosus
RibP	ribosomal P
RNP	ribonucleoprotein
SLE	systemic lupus erythematosus
Sm	Smith
SNP	single nucleotide polymorphism
SSA/B	Sjogren’s syndrome A/B
TNF	tumor necrosis factor

## References

[1] (1999). The American College of Rheumatology nomenclature and case definitions for neuropsychiatric lupus syndromes. Arthritis Rheum..

[2] Rubinstein T.B., Putterman C., Goilav B. (2015). Biomarkers for CNS involvement in pediatric lupus. Biomarkers Med..

[3] Jayasinghe M., Rashidi F., Gadelmawla A.F., Pitton Rissardo J., Rashidi M., Elendu C.C., Fornari Caprara A.L., Khalil I., Hmedat K.I., Atef M. (2025). Neurological manifestations of systemic lupus erythematosus: A comprehensive review. Cureus.

[4] Appenzeller S., Pereira D.R., Julio P.R., Reis F., Rittner L., Marini R. (2022). Neuropsychiatric manifestations in childhood-onset systemic lupus erythematosus. Lancet Child Adolesc. Health.

[5] Natoli V., Charras A., Hahn G., Hedrich C.M. (2023). Neuropsychiatric involvement in juvenile-onset systemic lupus erythematosus (jSLE). Mol. Cell Pediatr..

[6] Lindblom J., Mohan C., Parodis I. (2022). Biomarkers in neuropsychiatric systemic lupus erythematosus: A systematic literature review of the last decade. Brain Sci..

[7] Bărbulescu A.L., Sandu R.E., Vreju A.F., Ciurea P.L., Criveanu C., Firulescu S.C., Chisălău A.B., Pârvănescu C.D., Ciobanu D.A., Radu M. (2019). Neuroinflammation in systemic lupus erythematosus—A review. Rom. J. Morphol. Embryol..

[8] Kammeyer R., Chapman K., Furniss A., Hsieh E., Fuhlbrigge R., Ogbu E.A., Boackle S., Zell J., Nair K.V., Borko T.L. (2024). Blood-based biomarkers of neuronal and glial injury in active major neuropsychiatric systemic lupus erythematosus. Lupus.

[9] Landman A.J.E.M.C., Don E.E., Vissers G., Ket H.C.J., Oudijk M.A., de Groot C.J.M., Huirne J.A.F., de Boer M.A. (2022). Modified Newcastle Ottawa quality assessment scale and AHRQ standards. PLoS ONE.

[10] Schünemann H.J., Oxman A.D., Brozek J., Glasziou P., Jaeschke R., Vist G.E., Williams J.W., Kunz R., Craig J., Montori V.M. (2008). Grading quality of evidence and strength of recommendations for diagnostic tests and strategies. BMJ.

[11] Frittoli R.B., Pereira D.R., Lapa A.T., Postal M., Sinicato N.A., Fernandes P.T., Cendes F., Castellano G., Rittner L., Marini R. (2022). Axonal dysfunction is associated with interferon-γ levels in childhood-onset systemic lupus erythematosus: A multivoxel magnetic resonance spectroscopy study. Rheumatology.

[12] Labouret M., Trebossen V., Ntorkou A., Bartoli S., Aubart M., Auvin S., Bader-Meunier B., Baudouin V., Corseri O., Dingulu G. (2024). Juvenile neuropsychiatric systemic lupus erythematosus: A specific clinical phenotype and proposal of a probability score. Lupus.

[13] Labouret M., Costi S., Bondet V., Trebossen V., Le Roux E., Ntorkou A., Bartoli S., Auvin S., Bader-Meunier B., Baudouin V. (2023). Juvenile neuropsychiatric systemic lupus erythematosus: Identification of novel central neuroinflammation biomarkers. J. Clin. Immunol..

[14] DiFrancesco M.W., Gitelman D.R., Klein-Gitelman M.S., Sagcal-Gironella A.C., Zelko F., Beebe D., Parrish T., Hummel J., Ying J., Brunner H.I. (2013). Functional neuronal network activity differs with cognitive dysfunction in childhood-onset systemic lupus erythematosus. Arthritis Res. Ther..

[15] Gitelman D.R., Klein-Gitelman M.S., Ying J., Sagcal-Gironella A.C., Zelko F., Beebe D.W., Difrancesco M., Parrish T., Hummel J., Beckwith T. (2013). Brain morphometric changes associated with childhood-onset systemic lupus erythematosus and neurocognitive deficit. Arthritis Rheum..

[16] Jones J.T., DiFrancesco M., Zaal A.I., Klein-Gitelman M.S., Gitelman D., Ying J., Brunner H.I. (2015). Childhood-onset lupus with clinical neurocognitive dysfunction shows lower streamline density and pairwise connectivity on diffusion tensor imaging. Lupus.

[17] Tan E.M., Cohen A.S., Fries J.F., Masi A.T., McShane D.J., Rothfield N.F., Schaller J.G., Talal N., Winchester R.J. (1982). The 1982 revised criteria for the classification of systemic lupus erythematosus. Arthritis Rheum..

[18] Dong J., Li H., Wang J., Yao Y., Yang Q.R. (2012). Predictors for neuropsychiatric development in Chinese adolescents with systemic lupus erythematosus. Rheumatol. Int..

[19] Lapa A.T., Postal M., Sinicato N.A., Bellini B.S., Fernandes P.T., Marini R., Appenzeller S. (2017). S100b is associated with cognitive impairment in childhood-onset systemic lupus erythematosus patients. Lupus.

[20] Press J., Palayew K., Laxer R.M., Elkon K., Eddy A., Rakoff D., Silverman E.D. (1996). Antiribosomal P antibodies in pediatric patients with systemic lupus erythematosus and psychosis. Arthritis Rheum..

[21] Rana A., Minz R., Aggarwal R., Anand S., Pasricha N., Singh S. (2012). Gene expression of cytokines (TNF-α, IFN-γ), serum profiles of IL-17 and IL-23 in paediatric systemic lupus erythematosus. Lupus.

[22] Hochberg M.C. (1997). Updating the American College of Rheumatology revised criteria for the classification of systemic lupus erythematosus. Arthritis Rheum..

[23] Bao S., Huang H., Jin Y., Ding F., Yang Z., Xu X., Liu C., Lu J., Jin Y. (2023). Autoantibody-based subgroups and longitudinal seroconversion in juvenile-onset systemic lupus erythematosus. Lupus Sci. Med..

[24] Brunner H.I., Klein-Gitelman M.S., Zelko F., Beebe D.W., Foell D., Lee J., Zaal A., Jones J., Roebuck-Spencer T., Ying J. (2014). Blood-based candidate biomarkers of the presence of neuropsychiatric systemic lupus erythematosus in children. Lupus Sci. Med..

[25] Fathy H.A., Alkady M.M., Tawfik M.S. (2022). Tumor necrosis factor receptor 2 and anti-ribosomal P antibodies as biomarkers in juvenile neuropsychiatric systemic lupus erythematosus. J. Radiat. Res. Appl. Sci..

[26] Giani T., Smith E.M.D., Al-Abadi E., Armon K., Bailey K., Ciurtin C., Davidson J., Gardner-Medwin J., Haslam K., Hawley D.P. (2021). Neuropsychiatric involvement in juvenile-onset systemic lupus erythematosus: Data from the UK Juvenile-onset systemic lupus erythematosus cohort study. Lupus.

[27] Jurencák R., Fritzler M., Tyrrell P., Hiraki L., Benseler S., Silverman E. (2009). Autoantibodies in pediatric systemic lupus erythematosus: Ethnic grouping, cluster analysis, and clinical correlations. J. Rheumatol..

[28] Harel L., Sandborg C., Lee T., von Scheven E. (2006). Neuropsychiatric manifestations in pediatric systemic lupus erythematosus and association with antiphospholipid antibodies. J. Rheumatol..

[29] Khajezadeh M.A., Zamani G., Moazzami B., Nagahi Z., Mousavi-Torshizi M., Ziaee V. (2018). Neuropsychiatric involvement in juvenile-onset systemic lupus erythematosus. Neurol. Res. Int..

[30] Liphaus B.L., Silva S.C., Palmeira P., Silva C.A., Goldenstein-Schainberg C., Carneiro-Sampaio M. (2024). Reduced expressions of apoptosis-related proteins TRAIL, Bcl-2, and TNFR1 in NK cells of juvenile-onset systemic lupus erythematosus patients: Relations with disease activity, nephritis, and neuropsychiatric involvement. Front. Immunol..

[31] Moraitis E., Stathopoulos Y., Hong Y., Al-Obaidi M., Mankad K., Hacohen Y., Sen D., Hemingway C., Eleftheriou D. (2019). Aquaporin-4 IgG antibody-related disorders in patients with juvenile systemic lupus erythematosus. Lupus.

[32] Mostafa G.A., Ibrahim D.H., Shehab A.A., Mohammed A.K. (2010). The role of measurement of serum autoantibodies in prediction of pediatric neuropsychiatric systemic lupus erythematosus. J. Neuroimmunol..

[33] Nowling T.K., Kral M., Wolf B., Gilkeson G., Ruth N.M. (2021). Formal neurocognitive function and anti-N-methyl-D-aspartate receptor antibodies in paediatric lupus. Lupus Sci. Med..

[34] Rahman H.M., Hashim H.M., Karam R.A. (2012). Psychiatric disorders in juvenile systemic lupus erythematosus: Prevalence and association with autoantibodies. Middle East. Curr. Psychiatry.

[35] Singh S., Gupta M.K., Ahluwalia J., Singh P., Malhi P. (2009). Neuropsychiatric manifestations and antiphospholipid antibodies in pediatric onset lupus: 14 years of experience from a tertiary center of North India. Rheumatol. Int..

[36] Valões C.C., Molinari B.C., Pitta A.C., Gormezano N.W., Farhat S.C., Kozu K., Sallum A.M., Appenzeller S., Sakamoto A.P., Terreri M.T. (2017). Anti-ribosomal P antibody: A multicenter study in childhood-onset systemic lupus erythematosus patients. Lupus.

[37] Yu H.H., Lee J.H., Wang L.C., Yang Y.H., Chiang B.L. (2006). Neuropsychiatric manifestations in pediatric systemic lupus erythematosus: A 20-year study. Lupus.

[38] Zuniga Zambrano Y.C., Guevara Ramos J.D., Penagos Vargas N.E., Benitez Ramirez D.C., Ramirez Rodriguez S.M., Vargas Niño A.C., Izquierdo Bello A.H. (2014). Risk factors for neuropsychiatric manifestations in children with systemic lupus erythematosus: Case-control study. Pediatr. Neurol..

[39] Aringer M., Costenbader K., Daikh D., Brinks R., Mosca M., Ramsey-Goldman R., Smolen J.S., Wofsy D., Boumpas D.T., Kamen D.L. (2019). 2019 European League Against Rheumatism/American College of Rheumatology classification criteria for systemic lupus erythematosus. Arthritis Rheumatol..

[40] Shaaban Y.S., Hammed A.M.E., El-Sayed R.M. (2023). Aquaporin-4 IgG antibodies: Predictors of positivity and their relationship with neuropsychiatric disorders and white matter lesions in Juvenile systemic lupus erythematosus. Pediatr. Rheumatol..

[41] Soliman H., El-Kafafy E., Abdelaziz M. (2023). Hyperprolactinemia and its correlation with disease activity and neuropsychiatric manifestations in children with systemic lupus erythematosus. Pediatr. Rheumatol..

[42] Ye C., Chen L., Zhang L., Zheng Y., Liu X., Huang Z., Tang K., Jiang X., Chen P. (2025). IL-17A, IL-23R, FCGR3A are associated with neuropsychiatric systemic lupus erythematosus susceptibility in pediatric patients with lupus nephritis. Cytokine.

[43] Miyaue N., Yamanishi Y., Ito Y., Ando R., Nagai M. (2024). CSF Neopterin levels are elevated in various neurological diseases and aging. J. Clin. Med..

[44] Mahmoud R.A., El-Gendi H.I., Ahmed H.H. (2005). Serum neopterin, tumor necrosis factor-alpha and soluble tumor necrosis factor receptor II (p75) levels and disease activity in Egyptian female patients with systemic lupus erythematosus. Clin. Biochem..

[45] Tay S.H., Fairhurst A.M., Mak A. (2017). Clinical utility of circulating anti-N-methyl-d-aspartate receptor subunits NR2A/B antibody for the diagnosis of neuropsychiatric syndromes in systemic lupus erythematosus and Sjögren’s syndrome: An updated meta-analysis. Autoimmun. Rev..

[46] Mike E.V., Makinde H.M., Gulinello M., Vanarsa K., Herlitz L., Gadhvi G., Winter D.R., Mohan C., Hanly J.G., Mok C.C. (2019). Lipocalin-2 is a pathogenic determinant and biomarker of neuropsychiatric lupus. J. Autoimmun..

[47] Emerson J.S., Gruenewald S.M., Gomes L., Lin M.W., Swaminathan S. (2023). The conundrum of neuropsychiatric systemic lupus erythematosus: Current and novel approaches to diagnosis. Front. Neurol..

[48] Patel V. (2024). The challenge of neuropsychiatric systemic lupus erythematosus: From symptoms to therapeutic strategies. Diagnostics.

[49] Muscal E., Brey R.L. (2010). Neurologic manifestations of systemic lupus erythematosus in children and adults. Neurol. Clin..

[50] Mikdashi J., Handwerger B. (2004). Predictors of neuropsychiatric damage in systemic lupus erythematosus: Data from the Maryland lupus cohort. Rheumatology.

[51] Sanna G., Bertolaccini M.L., Cuadrado M.J., Laing H., Khamashta M.A., Mathieu A., Hughes G.R. (2003). Neuropsychiatric manifestations in systemic lupus erythematosus: Prevalence and association with antiphospholipid antibodies. J. Rheumatol..

[52] Ho R.C., Ong H., Thiaghu C., Lu Y., Ho C.S., Zhang M.W. (2016). Genetic variants that are associated with neuropsychiatric systemic lupus erythematosus. J. Rheumatol..

[53] Imani D., Rezaei R., Poorsheikhani A., Alizadeh S., Mahmoudi M. (2018). Association of IL-23R gene rs7517847 T>G SNP and susceptibility to systemic lupus erythematosus: A systematic review and meta-analysis. Rheumatol. Res..

[54] Padhi S., Sarangi S., Nayak N., Barik D., Pati A., Panda A.K. (2022). Interleukin 17A rs2275913 polymorphism is associated with susceptibility to systemic lupus erythematosus: A meta and trial sequential analysis. Lupus.

[55] Magro-Checa C., Steup-Beekman G.M., Huizinga T.W., van Buchem M.A., Ronen I. (2018). Laboratory and neuroimaging biomarkers in neuropsychiatric systemic lupus erythematosus: Where do we stand, where to go?. Front. Med..

[56] Sarbu N., Toledano P., Calvo A., Roura E., Sarbu M.I., Espinosa G., Lladó X., Cervera R., Bargalló N. (2017). Advanced MRI techniques: Biomarkers in neuropsychiatric lupus. Lupus.

