# Supplementary material for: Biomarkers in Pediatric Neuropsychiatric Systemic Lupus Erythematosus: A Systematic Review
Source: Life (Basel). 2025 Sep 15;15(9):1445. doi: 10.3390/life15091445 (PMC12471529; doi:10.3390/life15091445)
Supplement: Supplementary file 1 [file life-15-01445-s001.zip › Table S1.pdf]

Table S1. Characteristics of the 29 included studies.

Study	Country	Study Design	Study Sample	Biomarkers Studied	Measurement Method	Primary Outcomes
Bao et al. 2023 [23]	China	Retrospective, cohort, single-center	87 jSLE	anti-CL, anti-b2GP1, anti-dsDNA, anti-RNP, anti-ENA	Double immunodiffusion, ELISA	Autoantibody clusters and NPSLE/lupus nephritis
Brunner et al., 2014 [24]	US	Cohort, longitudinal	40 cSLE	NGAL, S100B, S100A8/9, anti-NR2, anti-RibP, anti-CL, anti-b2GP1, LA	ELISA	Blood-based biomarkers for cSLE, neurocognitive dysfunction
Difrancesco et al., 2013 [14]	US	Cross-sectional (two sites)	22 cSLE[a]	brain activation	fMRI	Brain activation and cognitive dysfunction
Dong et al., 2012 [18]	China	Retrospective, cohort, single-center	67 SLE	ANA, anti-ENA, anti-CL, anti-dsDNA	IIF, Counterimmunoelectrophoresis, ELISA	Predictors for NPSLE development
Fathy et al., 2022 [25]	Egypt	Case-control, single-center	60 SLE, 30 controls	sTNFR2, anti-RibP	ELISA	sTNFR2/anti-RibP and NPSLE
Frittoli et al., 2022 [11]	Brazil	Cross-sectional, single-center	86 cSLE, 71 controls	Cytokines (serum IFN-γ, TNF-α, IL-4, -5, -6, -10, -12 and -17), S100b, ANA, anti-dsDNA, anti-ENA, anti-CL, MRS metabolites	ELISA, IIF, proton MRS	Axonal dysfunction, cognitive impairment, cytokine associations
Giani et al., 2023 [26]	UK	Cohort, multicenter	428 jSLE	ANA, anti-Sm, anti-dsDNA, anti-SS-A/Ro and SS-B/La, anti-PL	NR	Demographic, clinical, and laboratory features of NP-SLE
Gitelman et al., 2013 [15]	US	Cross-sectional (two sites)	22 cSLE[a], 19 controls	changes in gray and white matter volumes	MRI brain morphometry	Association of gray/white matter changes with neurocognitive deficit

Study	Country	Study Design	Study Sample	Biomarkers Studied	Measurement Method	Primary Outcomes
Harel et al., 2006 [28]	US	Retrospective, cohort, two centers	106 pSLE	anti-PL	ELISA	Prevalence of NP symptoms in SLE and association with anti-PL Abs
Jones et al., 2015 [16[	US	Cross-sectional pilot, two sites	15 cSLE[a], 14 controls	White matter connectivity	diffusion-tensor imaging	White-matter connectivity changes in NCD
Jurencák et al., 2009 [27]	Canada	Cohort, prospective, single-center	156 pSLE	Anti-CL, ANA, anti-ENA, anti-dsDNA, anti-RibP, anti-U1RNP, anti-scl70, Jo-1	ELISA	Ethnic differences in autoAb profiles and clinical associations
Khajezadeh et al., 2018 [29]	Iran	Prospective, cross-sectional, single-center	146 pSLE	ANA, anti-dsDNA	NR	Incidence and features of NP impairment
Labouret et al., 2023 [13]	France	Retrospective, cohort, single-center	51 jSLE[b] (20 jNPSLE)	CSF neopterin, IFN-α, ANA, anti-ENA, anti-RNP, anti-RibP, anti-scl70, anti-CL, anti-dsDNA, anti-b2GP1	LC-MS/MS, digital ELISA (Simoa® platform), IHC (rat brain slices)	CSF biomarkers for jNPSLE
Labouret et al., 2024 [12]	France	Retrospective, cohort, single-center	39 jSLE	CSF neopterin, anti-RibP, anti-dsDNA, ANA, anti-ENA, anti-RNP, anti-scl70, anti-CL, anti-b2GP1	LC-MS/MS, IHC (rat brain slices)	Develop a probability score for jNPSLE diagnosis
Lapa et al., 2017 [19]	Brazil	Cross-sectional, single-center	71 cSLE, 53 controls	S100b, anti-CL, ANA, anti-dsDNA, anti-ENA	ELISA, IIF	S100b association with cognitive impairment and NP manifestations

Study	Country	Study Design	Study Sample	Biomarkers Studied	Measurement Method	Primary Outcomes
Liphaus et al., 2024 [30]	Brazil	Cross-sectional, single-center	36 jSLE, 13 JDM, 9 controls	anti-dsDNA; Fas, FasL, TRAIL, TNFR1, Bcl-2, Bax, Bim, and caspase-3 expressions in NK cells	ELISA, flow cytometry	Association of apoptosis-related protein expression in NK cells with disease activity parameters, nephritis, and NP involvement
Moraitis et al., 2019 [31]	UK	Cross-sectional, two centers	90 jSLE	AQP4-IgG, MOG-Abs, anti-dsDNA, ANA, anti-ENA, anti-PL	Cell-based assay for AQP4; not mentioned for other markers	AQP4-IgG and neurological involvement
Mostafa et al., 2010 [32]	Egypt	Case-control, follow-up, single-center	30 SLE, 30 controls	anti-ganglioside M1, anti-RibP, anti-CL, ANA, anti-dsDNA	ELISA, immunofluorescence	Predictive value for NPSLE and cognitive dysfunction
Nowling et al., 2021 [33]	US	Cross-sectional, single-center	24 SLE, 12 JIA (control)	NMDAR, anti-RibP	ELISA	NMDAR/RibP and neurocognitive function
Press et al., 1996 [20]	Canada	Cohort, case-control, single-center	79 SLE, 60 controls, 12 primary psychosis	Anti-RibP, anti-dsDNA	ELISA	Anti-RibP in SLE psychosis
Rahman et al., 2012 [34]	Egypt	Cross-sectional, follow-up, single-center	35 jSLE	anti-PL, ANA, anti-RibP, anti-dsDNA	ELISA, IIF	Psychiatric disorders and autoAbs
Rana et al., 2012 [21]	India	Retrospective, cohort, single-center	40 pSLE, 20 controls	Peripheral IFN-γ and TNF-α, IL-17, IL-23	RT-PCR, ELISA	Gene expression and serum expression association with organ manifestations
Shaaban et al., 2023 [40]	Egypt	Cross-sectional, single-center	90 jSLE	AQP4-IgG, anti-dsDNA, ANA, anti-CL	ELISA	AQP4-IgG, NP disorders, white matter lesions

Study	Country	Study Design	Study Sample	Biomarkers Studied	Measurement Method	Primary Outcomes
Singh et al., 2009 [35]	India	Retrospective, cohort, single-center	53 pSLE	anti-CL	ELISA	Occurrence of anti-PL Abs in pNPSLE
Soliman et al., 2023 [41]	Egypt	Case-control, single-center	40 SLE, 40 controls	Prolactin	ELISA	Prolactin and neurological manifestations
Valoes et al., 2017 [36]	Brazil	Retrospective, cohort, multicenter	228 cSLE	anti-RibP, ANA, anti-dsDNA, anti-ENA, anti-RNP, anti-CL	ELISA, IIF, counterimmunoelectrophoresis, passive hemagglutination	Compare clinical and disease features between anti-RibP-positive and negative patietns
Ye et al., 2025 [42]	China	Case-control, single-center	12 NPSLE/LN, 50 LN (controls)	54 SNPs in 20 genes	Agena MassARRAY platform	Risk of NPSLE in LN patients
Yu et al., 2006 [37]	China	Retrospective, cohort, single-center	185 pSLE	anti-dsDNA, anti-CL, anti-ENA, anti-PL	ELISA	Manifestations, treatment, and outcome of NP involvement in SLE
Zambrano et al., 2014 [38]	Colombia	Retrospective, case-control, single-center	90 pSLE (30 with NP, 60 non-NP controls)	anti-dsDNA, ANA, anti-CL, anti-b2GP1, anti-ENA	NR	Risk factors associated with NPSLE

[a] The studies by Difrancesco et al., Gitelman et al., and Jones et al. included subsets of patients re-cruited in a larger study. Given the similar patient characteristics, publication years, and common authors, the included patients may be the same across the three studies.

[b] Includes the 39 patients analyzed in the study by Labouret et al. 2024.

Notes: Anti-ENA include anti-SM, anti-SSA, and anti-SSB. Studies are listed alphabetically.

Abbreviations: Ab, antibody; ANA, antinuclear antibodies; AQP4,, aquaporin 4; CL, cardiolipin; CSF, cerebrospinal fluid; cSLE, childhood-onset SLE; ds, double-stranded; ELISA, enzyme-linked immunoabsorbent assay; ENA, extractable nuclear antigens; fMRI, functional MRI; GP1, glycoprotein 1; IFN, interferon; IHC, immunohistochemistry; IIF, indirect immunofluorescence; IL, interleukin; jSLE, juvenile-onset SLE; LC-MS/MS, liquid chromatography coupled to tandem mass spectrometry; LN, lupus nephritis; MOG, myelin oligodendrocyte glycoprotein; MRI, magnetic resonance imaging; MRS, magnetic resonance spectroscopy; NCD, neurocognitive dysfunction; NGAL, neutrophil gelatinase associated lipocalin; NMDA, N-methyl-D-aspartate; NMDAR, NMDA receptor; NP, neuropsychiatric; NR2, NMDA receptor 2; PL,

phospholipid; pNPSLE, pediatric-onset NPSLE; pSLE, pediatric-onset SLE; RibP, ribosomal P; RNP, ribonucleoprotein; S100, S100 calcium binding protein; scl70, topoisomerase I; SLE, systemic lupus erythematosus; SNP, single nucleotide polymorphism; SSA/B, Sjogren's syndrome antibodies A/B; sTNFR2, soluble TNF receptor 2; TNF, tumor necrosis factor; U1RNP, U1 ribonucleoprotein.
