# Supplementary material for: Biomarkers in Pediatric Neuropsychiatric Systemic Lupus Erythematosus: A Systematic Review
Source: Life (Basel). 2025 Sep 15;15(9):1445. doi: 10.3390/life15091445 (PMC12471529; doi:10.3390/life15091445)
Supplement: Supplementary file 1 [file life-15-01445-s001.zip › Table S2.pdf]

Table S2. Patient and Disease Characteristics.

Study	pSLE (n)	NPSLE total (n)	NPSLE at diagnosis (n)	Age at enrolment (years)	Age at SLE diagnosis (years)	Female sex n (%)	Neuropsychiatric manifestations (%)[a]
Bao et al. 2023 [23]	87	22	22	NR	2.75–17.75, mean: 11.71	78 (90.3%)	NR
Brunner et al., 2014 [24]	40	9	NR	8–19	<16	34 (85%)	NCD (100%)
Difrancesco et al., 2013 [14]	22[b]	8	NR	9–18	<16	17 (77.3%)	NCD (100%)
Dong et al., 2012 [18]	67	24	9	NR	10–18, mean at NPSLE onset: 14.42	58 (86.6%)	cognitive dysfunction (50%), seizure (43.3%), headache (30%), psychosis, myelopathy, mood disorder, CVD, cranial neuropathy, mononeuropathy multiplex (occurrence not reported)
Fathy et al., 2022 [25]	60	30	NR	6–16, mean 12.7	<18	38 (63.3%)	headaches, mood disturbances, psychosis, depression, cognitive dysfunction, seizures
Frittoli et al., 2022 [11]	86	NR	NR	5–28, median: 17	<18	76 (88.4%)	headache (62.8%), psychosis (34.8%), cognitive dysfunction (22.1%), seizure (17.4%), CVD (10.5%), depression (9.3%)
Giani et al., 2023 [26]	428	107	52	NR	0.1–17.9, mean 12.2	90 (84.7%)	headache (78.5%), mood disorder (48.6%), cognitive dysfunction (42%), anxiety (23.3%), seizure (19.6%), movement disorder (17.7%), CVD (14.9%), psychosis (9.3%), mononeuropathy (8.4%), acute confusional state (6.5%), cranial neuropathy (5.6%)

Study	pSLE (n)	NPSLE total (n)	NPSLE at diagnosis (n)	Age at enrolment (years)	Age at SLE diagnosis (years)	Female sex n (%)	Neuropsychiatric manifestations (%)[a]
Gitelman et al., 2013 [15]	22[b]	8	NR	9–18, mean: 14.9	<16	18 (82%)	NCD (100%)
Harel et al., 2006 [28]	106	25	12	NR	5–18, mean: 12.3	81 (76%)	seizures (40%), headache, mood disorder (depression), cognitive dysfunction (20% each), cerebrovascular accident, psychosis, pseudomotor cerebri (12% each)
Jones et al., 2015 [16[	15[b]	6	NR	9–17	<16	12 (80%)	NCD (100%), neuropsychiatric (16.7%)
Jurencák et al., 2009 [27]	156	35	35[c]	NR	<18, mean 12.6	130 (83.4%)	psychosis (31.4%), CVD (28.6%), headaches (14.3%), seizures (11.4%), cognitive dysfunction (8.6%), mood disorder (5.7%)
Khajezadeh et al., 2018 [29]	146	41	18	mean 12.2	<18, mean 10.2	110 (75.3%)	Headache (46.3%), cognitive disorder (41.5%), seizures (34.1%), psychiatric disorder (31.7%), mood disorder (19.5%), psychosis (7.3%), CVD (5.3% of SLE)
Labouret et al., 2023 [13]	51[d]	20	13	<18	<16, median 14.0 for pNPSLE	19 (95% of pNPSLE)	mood disorder (90%), cognitive symptoms (80%), sleep disorder (80%), neurologic/motor symptoms (60%), headache (45%)
Labouret et al., 2024 [12]	39	17	10	NR	<16, median 13.4	34 (87%)	cognitive dysfunction (82%), psychosis (76%), anxiety disorder (71%), headache (53%), mood disorder (35%), acute confusional state (18%), aseptic meningitis (12%), seizures (6%)

Study	pSLE (n)	NPSLE total (n)	NPSLE at diagnosis (n)	Age at enrolment (years)	Age at SLE diagnosis (years)	Female sex n (%)	Neuropsychiatric manifestations (%)[a]
Lapa et al., 2017 [19]	71	58	NR	9–37, median: 18	<18	67 (94.4%)	headache (72.4%), cognitive dysfunction (48.3%), anxiety (37.9%), depression (25.9%), seizure (24.1%), psychosis (8.6%), acute confusional state (5.1%)
Liphaus et al., 2024 [30]	36	9	NR	7.1–20.3, median: 15.8	4.1–18.7, median: 10.9	31 (86.1%)	NS
Moraitis et al., 2019 [31]	90	28	NR	11–27, median: 18.7	0.3–15, median: 8	79 (87.8%)	Neurological manifestations (100%)
Mostafa et al., 2010 [32]	30	12	0	8–16, mean: 11.6	<16	24 (80%)	cognitive dysfunction (83.3%), depression (58.3%), pyramidal tract lesions (33.3%), transient ischemic attacks (25%), anxiety (16.7%), cranial lesions (16.7%), seizures (16.7%), peripheral neuritis (8.3%)
Nowling et al., 2021 [33]	24	17 (1)[e]	NR	mean: 15.4	<18	20 (83.3%)	cognitive deficits (100%), acute psychosis (1 patient)
Press et al., 1996 [20]	79	13	NR	<18	<18	NR	psychosis, depression
Rahman et al., 2012 [34]	35	23	13	7–16, mean: 12.8	<16	30 (85.7%)	anxiety (69.6%), depression (43.5%), psychosis (8.7%)
Rana et al., 2012 [21]	40	10	NR	5-16, median: 12	<16	34 (85%)	CNS manifestations (100%)

Study	pSLE (n)	NPSLE total (n)	NPSLE at diagnosis (n)	Age at enrolment (years)	Age at SLE diagnosis (years)	Female sex n (%)	Neuropsychiatric manifestations (%)[a]
Shaaban et al., 2023 [40]	90	59	NR	mean: 14.46	<18	83 (92.2)%	mood disorders (57.8%), psychosis (50%), headache (47.8%), anxiety (47.8%), seizures (31.1%), CVD (26.7%), polyneuropathy (17.8%), acute confusional state (12.2), movement disorder (10%), cranial neuropathy (10%), cognitive dysfunction (8.9%), aseptic meningitis (7.8%), autonomic neuropathy (5.6%)
Singh et al., 2009 [35]	53	27	15	mean: 9.9	<18	39 (73.6%)	headache (77.8%), seizures (70.4%), cognitive dysfunction (33.3%), CVD (22.2%), psychosis and mood disorders (18.5% each), acute confusional state (14.8%), movement disorder and PNS (11.1%, each), aseptic meningitis (7.4%)
Soliman et al., 2023 [41]	40	7	NR	<16, mean: 11.5	<16	34 (85.0%)	NS
Valoes et al., 2017 [36]	228	120	NR	2–25.3	<18	199 (87.3%)	headache (64.2%), seizures (31.7%), psychosis (23.3%, mood disorders (20.8%), PNS (10%), acute confusional state (13%), cognitive dysfunction (8.3%), anxiety (7.5%), movement disorder (chorea; 5%)
Ye et al., 2025 [42]	62	12	3	Mean: 12.34	<18	54 (87.1%)	headache, anxiety, seizures

Study	pSLE (n)	NPSLE total (n)	NPSLE at diagnosis (n)	Age at enrolment (years)	Age at SLE diagnosis (years)	Female sex n (%)	Neuropsychiatric manifestations (%)[a]
Yu et al., 2006 [37]	185	64	14	NR	SLE: 4.3–18, mean: 13.2<br>NPSLE: 5–28.7, mean: 15.2	158 (85.4%)	seizures (84.4%), CVD (39%), psychosis (21.9%), depression 12.5%), acute confusional state (12.5%), headache (10.9%), anxiety disorder (6.3%)
Zambrano et al., 2014 [38]	90	30	2	3–17, mean: 12.2	<18	76 (84.4%)	Seizures (50%); headache, depression (36% each), CVA (26%); vasculitis, chorea (16% each); psychosis, neuropathy (13% each), myelitis (6%)

[a] The most common reported manifestations are included, i.e., present in >5% of the patients with NPSLE. If available, the prevalence of each manifestation is included in parenthesis as a percentage of NPSLE (or SLE in the studies by Frittoli et al. and Shaaban et al. where subgroup analysis was not performed).

[b] The studies by Difrancesco et al., Gitelman et al., and Jones et al. included subsets of patients recruited in a larger study. Given the similar patient characteristics, publication years, and common authors, the included patients may be the same across the three studies.

[c] At diagnosis or within the 1st year after

[d] Includes the 39 patients analyzed in the study by Labouret et al. 2024.

[e] One patient was reported to have CNS involvement at enrolment, which was diagnosed as acute psychosis; 17/24 SLE patients were found to have modest cognitive performance in a battery of neurocognitive tests.

Note: Studies are listed alphabetically

Abbreviations: CNS, central nervous system; CVD, cerebrovascular disease; NCD, neurocognitive deficit; NPSLE, neuropsychiatric SLE; NR, not reported; NS, not specified; PNS, peripheral nervous system, pSLE, pediatric-onset SLE; SLE, systemic lupus erythematosus
