# Supplementary material for: Biomarkers in Pediatric Neuropsychiatric Systemic Lupus Erythematosus: A Systematic Review
Source: Life (Basel). 2025 Sep 15;15(9):1445. doi: 10.3390/life15091445 (PMC12471529; doi:10.3390/life15091445)
Supplement: Supplementary file 1 [file life-15-01445-s001.zip › Table S3.pdf]

Table S3. Newcastle-Ottawa Scale Scoring of Studies and Quality Assessment as per the AHRD Standards

Study Name (First Author & Year)[a]	Study Type[b]	Selection (Score/Max Stars)	Comparability (Score/Max Stars)	Outcome/Exposure (Score/Max Stars)	Total Stars	Quality Assessment (AHRD)[c]
Bao et al. 2023 [23]	Cohort	2/4	0/2	3/3	5	Poor
Brunner et al., 2014 [24]	Cohort	3/4	2/2	2/3	7	Good
Difrancesco et al., 2013 [14]	Cross-Sectional	1/3	1/2	2/2	4	Poor
Dong et al., 2012 [18]	Cohort	1/4	0/2	2/3	3	Poor
Fathy et al., 2022 [25]	Case-Control	3/4	1/2	2/3	6	Good
Frittoli et al., 2022 [11]	Cross-Sectional	1/3	1/2	2/2	4	Poor
Giani et al., 2023 [26]	Cohort	2/4	0/2	2/3	4	Poor
Gitelman et al., 2013 [15]	Cross-Sectional	1/3	2/2	2/2	5	Poor
Harel et al., 2006 [28]	Cohort	2/4	0/2	2/3	4	Poor
Jones et al., 2015 [16[	Cohort	2/4	0/2	2/3	4	Poor
Jurencák et al., 2009 [27]	Cross-Sectional	1/3	1/2	2/2	4	Poor
Khajezadeh et al., 2018 [29]	Cross-Sectional	1/3	0/2	2/2	3	Poor
Labouret et al., 2023 [13]	Cohort	1/4	0/2	2/3	3	Poor
Labouret et al., 2024 [12]	Cohort	1/4	1/2	2/3	4	Poor
Lapa et al., 2017 [19]	Cross-Sectional	1/3	1/2	2/2	4	Poor
Liphaus et al., 2024 [30]	Cross-Sectional	1/3	0/2	2/2	3	Poor
Moraitis et al., 2019 [31]	Cross-Sectional	1/3	0/2	2/2	3	Poor
Mostafa et al., 2010 [32]	Case-Control	2/4	1/2	2/3	5	Fair
Nowling et al., 2021 [33]	Cross-Sectional	1/3	1/2	2/2	4	Poor
Press et al., 1996 [20]	Case-Control	2/4	1/2	2/3	5	Fair
Rahman et al., 2012 [34]	Cross-Sectional	1/3	0/2	2/2	3	Poor

Study Name (First Author & Year)[a]	Study Type[b]	Selection (Score/Max Stars)	Comparability (Score/Max Stars)	Outcome/Exposure (Score/Max Stars)	Total Stars	Quality Assessment (AHRD)[c]
Rana et al., 2012 [21]	Cross-Sectional	1/3	2/2	2/2	5	Poor
Shaaban et al., 2023 [40]	Cross-Sectional	2/3	1/2	2/2	5	Fair
Singh et al., 2009 [35]	Cohort	3/4	0/2	3/3	6	Poor
Soliman et al., 2023 [41]	Case-Control	3/4	1/2	2/3	6	Good
Valoes et al., 2017 [36]	Cohort	3/4	0/2	2/3	5	Poor
Ye et al., 2025 [42]	Case-Control	3/4	1/2	2/3	6	Good
Yu et al., 2006 [37]	Cohort	1/4	0/2	3/3	4	Poor
Zambrano et al., 2014 [38]	Case-Control	2/4	0/2	3/3	5	Poor

AHRD, Agency for Healthcare Research and Quality

a Studies are listed alphabetically

b All studies were classified as case-control, cohort, or cross-sectional based on the source information. See also Table S1.

c The following are accepted thresholds for converting the Newcastle-Ottawa scales to AHRQ standards (good, fair, and poor quality studies):

For cohort and case-control studies:

- "Good quality: 3 or 4 stars in selection domain AND 1 or 2 stars in comparability domain AND 2 or 3 stars in outcome/exposure domain"
- "Fair quality: 2 stars in selection domain AND 1 or 2 stars in comparability domain AND 2 or 3 stars in outcome/exposure domain"
- "Poor quality: 0 or 1 star in selection domain OR 0 stars in comparability domain OR 0 or 1 stars in outcome/exposure domain"

For cross-sectional studies:

- "Good quality: 4 or 5 stars in selection domain AND 1 or 2 stars in compatibility domain AND 2 or 3 stars in outcome/exposure domain"
- "Fair quality: 2 or 3 stars in selection domain AND 1 or 2 stars in comparability domain AND 2 or 3 stars in outcome/exposure domain"
- "Poor quality: 0 or 1 star in selection domain OR 0 stars in comparability domain OR 0 or 1 stars in outcome/exposure domain"
