# Supplementary figures and images for: Biomarkers in Pediatric Neuropsychiatric Systemic Lupus Erythematosus: A Systematic Review
Source: Life (Basel). 2025 Sep 15;15(9):1445. doi: 10.3390/life15091445 (PMC12471529; doi:10.3390/life15091445)

## Identification of studies via database search

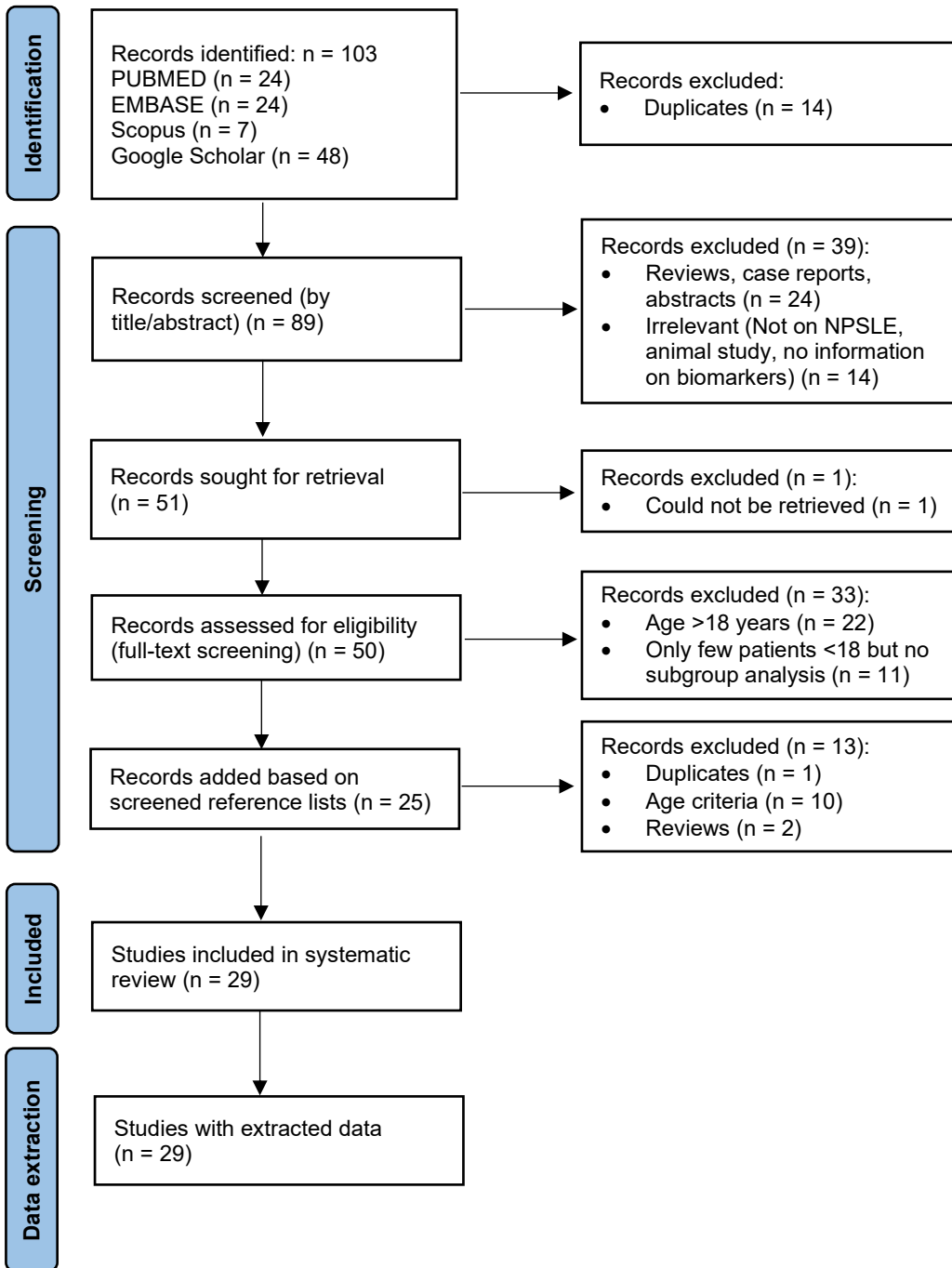

Supplement: Supplementary file 1 [file life-15-01445-s001.zip › Figure--PRISMA_flow_diagram.pdf]
